# Supplementary material for: Association between physical intimate partner violence and postpartum contraceptive use in the United States–evidence from PRAMS 2016–2021
Source: PLoS One. 2024 Dec 11;19(12):e0314938. doi: 10.1371/journal.pone.0314938 (PMC11633987; doi:10.1371/journal.pone.0314938)
Supplement: S1 Table — (DOCX) [file pone.0314938.s001.docx]

Annex

S1 Table: PRAMS, Phase 8 (2016-2021) Participating States according to data collection years

| STATE NAME | 2016  n (%) | 2017  n (%) | 2018  n (%) | 2019  n (%) | 2020  n (%) | 2021  n (%) | Total  n (%) |
| --- | --- | --- | --- | --- | --- | --- | --- |
| AK | 741 (3.2) | 1,065 (2.86) | 1,005 (2.4) | 950 (2.16) | 1,023 (2.43) | 363 (2.66) | 5,147 (2.55) |
| AL | - | 578 (1.55) | 826 (1.98) | 757 (1.72) | 669 (1.59) | 269 (1.97 ) | 3,099 (1.53) |
| AR | 541 (2.33) | 358 (0.96) | 407 (0.97) | 1,005 (2.28) | 1,018 (2.42) | 297 (2.18) | 3,626 (1.8) |
| AZ | - | - | - | - | 508 (1.21) | 249 (1.83) | 757 (0.37) |
| CO | 1,069 (4.61) | 1,273 (3.42) | 1,079 (2.58) | 1,033 (2.34) | 1,103 (2.62) | 242 (1.77) | 5,799 (2.87) |
| CT | 757 (3.27) | 1,446 (3.89) | 1,500 (3.59) | 1,174 (2.66) | 1,313 (3.12) | 433 (3.17) | 6,623 (3.28) |
| DC | - | - | 323 (0.77) | 477 (1.08) | 562 (1.34) | 192 (1.41) | 1,554 (0.77) |
| DE | 667 (2.88) | 830 (2.23) | 847 (2.03) | 887 (2.01) | 909 (2.16) | 248 (1.82) | 4,388 (2.17) |
| FL | - | - | - | 590 (1.34) | 1,165 (2.77) | 367 (2.69) | 2,122 (1.05) |
| GA | - | 616 (1.66) | 840 (2.01) | 730 (1.66) | 735 (1.75) | 259 (1.9) | 3,180 (1.57) |
| HI | 748 (3.23) | 322 (0.87) | - | 197 (0.45) | 1,203 (2.86) | 393 (2.88) | 2,863 (1.42) |
| IA | 619 (2.67) | 973 (2.62) | 868 (2.08) | 872 (1.98) | 701 (1.67) | 259 (1.9) | 4,292 (2.12) |
| IL | 901 (3.89) | 1,246 (3.35) | 1,303 (3.12) | 1,238 (2.81) | 1,218 (2.89) | 398 (2.92) | 6,304 (3.12) |
| IN | - | - | 593 () | 240 (0.54) | - | - | 833 (0.41) |
| KS | - | 677 (1.82) | 969 (2.32) | 1,010 (2.29) | 1,104 (2.62) | 369 (2.71) | 4,129 (2.04) |
| KY | - | 505 (1.36) | 712 (1.7) | 876 (1.99) | 523 (1.24) | 242 (1.77) | 2,858 (1.41) |
| LA | 569 (2.45) | 867 (2.33) | 876 (2.1) | 1,031 (2.34) | 710 (1.69) | 254 (1.86) | 4,307 (2.13) |
| MA | 902 (3.89) | 1,396 (3.75) | 1,428 (3.42) | 1,635 (3.71) | 1,435 (3.41) | 459 (3.37) | 7,255 (3.59) |
| MD | 796 (3.43) | 1,039 (2.79) | 1,054 (2.52) | 923 (2.09) | 970 (2.3) | 361 (2.65) | 5,143 (2.55) |
| ME | 532 (2.3) | 837 (2.25) | 856 (2.05) | 825 (1.87) | 865 (2.06) | 297 (2.18) | 4,212 (2.09) |
| MI | 1,100 (4.75) | 1,903 (5.12) | 1,845 (4.41) | 1,477 (3.35) | 1,448 (3.44) | 442 (3.24) | 8,215 (4.07) |
| MN | - | - | 860 (2.06) | 1,085 (2.46) | 695 (1.65) | 164 (1.2) | 2,804 (1.39) |
| MO | 687 (2.96) | 1,108 (2.98) | 929 (2.22) | 1,283 (2.91) | 1,151 (2.73) | 259 (1.9) | 5,417 (2.68) |
| MS | - | - | 764 (1.83) | 1,270 (2.88) | 1,009 (2.4) | 415 (3.04) | 3,458 (1.71) |
| MT | - | 588 (1.58) | 848 (2.03) | 749 (1.7) | 588 (1.4) | 374 (2.74) | 3,147 (1.56) |
| NC | - | 680 (1.83) | 933 (2.23) | 868 (1.97) | 277 (0.66) | - | 2,758 (1.37) |
| ND | - | 398 (1.07) | 801 (1.92) | 737 (1.67) | 745 (1.77) | 263 (1.93) | 2,944 (1.46) |
| NE | 824 (3.55) | 480 (1.29) | 797 (1.91) | 1,568 (3.56) | 1,571 (3.73) | 496 (3.64) | 5,736 (2.84) |
| NH | 404 (1.74) | 534 (1.44) | 587 (1.4) | 645 (1.46) | 688 (1.63) | 141 (1.03) | 2,999 (1.48) |
| NJ | 854 (3.68) | 1,222 (3.28) | 1,211 (2.9) | 1,070 (2.43) | 1,130 (2.68) | 365 (2.68) | 5,852 (2.9) |
| NM | 675 (2.91) | 1,281 (3.44) | 1,162 (2.78) | 1,198 (2.72) | 1,083 (2.57) | 329 (2.41) | 5,728 (2.84) |
| NY | 614 (2.65) | 881 (2.37) | 795 (1.9) | 751 (1.7) | 245 (0.58) | - | 3,286 (1.63) |
| OK | 1,113 (4.8) | 1,670 (4.49) | 1,491 (3.57) | 579 (1.31) | - | - | 4,853 (2.4) |
| OR | - | - | 974 (2.33) | 2,167 (4.92) | 1,918 (4.56) | 664 (4.87) | 5,723 (2.83) |
| PA | 697 (3.01) | 1,133 (3.05) | 1,041 (2.49) | 1,081 (2.45) | 1,079 (2.56) | 374 (2.74) | 5,405 (2.68) |
| PR | - | 521 (1.4) | 1,056 (2.53) | 1,034 (2.35) | 898 (2.13) | 364 (2.67) | 3,873 (1.92) |
| RI | 749 (3.23) | 1,060 (2.85) | 1,105 (2.64) | 1,041 (2.36) | 318 (0.76) | - | 4,273 () |
| SD | - | 715 (1.92) | 1,063 (2.54) | 1,031 (2.34) | 987 (2.35) | 240 (1.76) | 4,036 (2) |
| TN | - | - | - | 415 (0.94) | 643 (1.53) | 180 (1.32) | 1,238 (0.61) |
| TX | 1,229 (5.3) | 587 (1.58) | - | - | - | - | 1,816 (0.9) |
| UT | 896 (3.87) | 1,469 (3.95) | 1,346 (3.22) | 1,574 (3.57) | 1,481 (3.52) | 451 (3.31) | 3.57 |
| VA | 425 (1.83) | 941 (2.53) | 1,063 (2.54) | 1,051 (2.38) | 912 (2.17) | 286 (2.1) | 4,678 (2.32) |
| VT | 599 (2.58) | 851 (2.29) | 835 (2) | 761 (1.73) | 768 (1.82) | 204 (1.5) | 4,018 (1.99) |
| WA | 814 (3.51) | 1,285 (3.45) | 1,149 (2.75) | 1,161 (2.63) | 1,194 (2.84) | 440 (3.23) | 6,043 (2.99) |
| WI | 839 (3.62) | 1,276 (3.43) | 1,145 (2.74) | 910 (2.06) | 1,467 (3.49) | 468 (3.43) | 6,105 (3.02) |
| WV | 500 (2.16) | 688 (1.85) | 768 (1.84) | 222 (0.5) | 362 (0.86) | 233 (1.71) | 2,773 (1.37) |
| WY | 417 (1.8) | 577 (1.55) | 451 (1.08) | 548 (1.24) | 476 (1.13) | 143 (1.05) | 2,612 (1.29) |
| YC | 901 (3.89) | 1,324 (3.56) | 1,305 (3.12) | 1,354 (3.07) | 1,220 (2.9) | 393 (2.88) | 6,497 (3.22) |
| **Total** | **23,179** | **37,200** | **41,810** | **44,080** | **42,087** | **13,639** | **201,995** |
