# Supplementary material for: Association between physical intimate partner violence and postpartum contraceptive use in the United States–evidence from PRAMS 2016–2021
Source: PLoS One. 2024 Dec 11;19(12):e0314938. doi: 10.1371/journal.pone.0314938 (PMC11633987; doi:10.1371/journal.pone.0314938)
Supplement: Attachment — Submitted filename: Review for PLOS One.docx [file pone.0314938.s002.docx]

Review for PLOS One

1. Suggest clarifying that this paper is about physical IPV in the title, as PRAMS also measures emotional and sexual IPV: “Association between intimate partner violence and postpartum contraceptive use in the United States– Evidence from PRAMS, 2016-2020”

Abstract

2. Please be very clear on what type of IPV is the focus of this paper and please be consistent throughout with terminology. There is switching from IPV to physical IPV. I suggest also using the term physical IPV.
3. PRAMS uses the terminology phases, rather than rounds. It should be PRAMS Phase 8.
4. Why was 2021 data not included? That was released in April 2023.
5. “Our sample included women who answered questions about the experience of physical IPV during and/or 12 months prior to their most recent pregnancy” – it’s not actually the most recent pregnancy. It’s the index birth from which the birth certificate was sampled. Someone could have had a pregnancy between the last birth and the timing of the survey.
6. “modern or natural method” how are you defining these categories? Why not just say use of any method?
7. This paper did not look at screening for physical IPV during pregnancy and/or its relationship to postpartum contraceptive use. The authors, however, suggest that “it is crucial to prioritize meeting unmet contraceptive needs as well as conducting universal screening for IPV among pregnant women.” Please align conclusions with the actual findings from this paper.

Introduction

8. “Research in the 1990s found that between 3% to 62 9% of pregnant women were estimated to have experienced IPV during pregnancy in the US, though the 63 real number could have been much higher than reported [13].” Why did the authors use this citation? These numbers were recently updated using PRAMS data…
   a. Please see: D'Angelo DV, Bombard JM, Lee RD, Kortsmit K, Kapaya M, Fasula A. Prevalence of Experiencing Physical, Emotional, and Sexual Violence by a Current Intimate Partner during Pregnancy: Population-based Estimates from the Pregnancy Risk Assessment Monitoring System. J Fam Violence. 2022 Jan 14;38(1):117-126. doi: 10.1007/s10896-022-00356-y. PMID: 37205924; PMCID: PMC10193455.
9. “Mistimed or unwanted pregnancies can lead to induced abortion; an estimated 40% of unintended pregnancies end in abortion in the US [17]. Increases in correct and consistent contraceptive use, especially among populations at increased risk of having an unmet need for contraception, are therefore likely to lead to a decline in induced abortions.” Perhaps… but is this really a concern/goal? Induced abortion is not an adverse outcome and having an induced abortion could represent someone exerting their autonomy to do so and their reproductive preferences. Further, contraceptive use, alone, is not the solution to preventing unintended pregnancy. It is one tool that people can use to prevent pregnancy if that is their desire. I think this paper could use a good review of the framing to use more health equity/person centered language.
10. “However, to our knowledge, no recent studies have aimed at understanding the extent of the relationship of IPV on women’s contraceptive use in the US.” This is not true. The PRAMS team published an almost identical paper with data from Phase 7.
    a. Stevenson AA, Bauman BL, Zapata LB, Ahluwalia IB, Tepper NK. Intimate Partner Violence around the Time of Pregnancy and Postpartum Contraceptive Use. Womens Health Issues. 2020 Mar-Apr;30(2):98-105. doi: 10.1016/j.whi.2019.11.006. Epub 2020 Jan 5. PMID: 31911042.

Methods

11. PRAMS is implemented in collaboration with state and local health departments
12. PRAMS isn’t always state level. NYC, DC and Puerto Rico are also included in Phase 8.
13. “These are the most recent available data containing information on IPV and contraceptive use behavior for women of reproductive age in the US” Why wasn’t 2021 data incorporated into this analysis? Then this sentence would be true.
14. Again, please use the terminology that PRAMS prefer – Phase 8
15. To clarify, PRAMS does not collect data on gender identity. Many PRAMS publications use the term “women”, so I think it’s okay. However, a limitation is that we don’t actually know the gender identity of people included in this analysis, only that they were listed as the birthing parent on the birth certificate. So it is possible that people who do not identify as cisgender women were included and they are being misgendered by the terms used in this paper.
16. It’s actually 2 – 6 months after delivery that people were mailed a survey.
17. The introduction and justification for the study focuses heavily on use of contraception as a means to avoid unintended pregnancy. However, the authors did not restrict the study sample to only people who wanted to avoid a next pregnancy. I suggest that the authors either do this, or add additional justification on why we would want people who want to achieve pregnancy use contraception. I think the authors are assuming this is the case, as the sample is people who gave birth in the prior 2-6 months, but many people in the US (30%) have interpregnancy intervals shorter than the recommended 18 months (although, 6 months is really the most adverse). I would suggest reframing in terms of why it is important women avoid short interpregnancy intervals.
18. PRAMS requires a minimum overall response rate threshold for the release of data for each year. Did all sites have data for all years included in this study? This should be specified. Some states only have data released for specific years, or even no years. So not all sites are included for every year available.
19. Why was “other” contraceptive methods recoded? There are responses that do actually fit within the other categories because of data collection errors. I suggest recoding as many as possible.
20. A huge limitation of using the method question is that lactational amenorrhea method (LAM) is not included. LAM does afford a good amount protection from pregnancy. You will find some responses like this in the “other” response category.
21. “This study does not have the scope to measure IPV beyond physical IPV as this information was not collected by the PRAMS core questions.” This is a study limitation; please move to discussion. Data is included on sexual and emotional IPV before, during, and after pregnancy, as well as physical IPV after pregnancy, but these are in the standard question set. You could have included this if you wanted to look at these associations.
22. What was the justification for grouping together AI/AN, Asian, etc in “other”? From Table 1, 25,242 people were included in this group. A best practice is to maintain a minimum set of categories (American Indian or Alaska Native, Asian, Black or African American, Native Hawaiian or Other Pacific Islander, and White), except when the sample size is so small that estimates would be unreliable.
23. How were people classified if they selected more than one race?
24. “The variable ‘state of residence’ was aggregated into four geographic 148 regions according to US Census divisions: Northeast, Midwest, South, and West [25].” Why? This likely absolves any association we would see between state of residence and access to contraception in the postpartum period.
25. Why wasn’t insurance status included as a covariate?

**Results**

26. “This contraceptive method-mix was calculated according to the efficacy of the last used method reported during the survey as described in the methods section.” This doesn’t need to be repeated.
27. Typo in this sentence: For the long-acting-reversible and permanent methods (LARC-PM) IUD and vasectomy use were hinger among IVP non-exposed group,”
28. “we found that women who had exposure to physical IPV in the last twelve months had 42% lower odds” This phrasing is incorrect. It’s people who experienced IPV in the 12 months prior to pregnancy and during pregnancy. It’s not the last 12 months.
29. “The association between IPV and contraceptive use remained the same for modern contraceptive use only which was tested separately (results not shown).” I think a better comparison would be methods that require prescription/interaction with a facility/provider/etc and those that don’t. A likely explanation is that people who experience violence may not seek healthcare from a provider (either due to fears of confidentiality, lack of trauma informed/high quality care, barriers to getting care, etc), and so they might use methods that don’t require a prescription or interaction with the healthcare system. HOWEVER, some of the methods that the authors classified as “natural” require partner support… periodic abstinence, withdrawal, etc. But people using these methods might not use consistently or might not be as exposed to sex as people not experiencing violence. I assume that breastfeeding and sexual frequency might also differed between the groups, and these are factors for pregnancy… and these were not controlled for.
30. Table 2: “Natural contraceptive methods are rhythm, withdrawal, abstinence, other etc.” Please clarify what is “etc”.
31. Authors should clarify all abbreviations in tables with a note (e.g., IUD)
32. “A main difference between women exposed to IPV and women who weren’t was in the use of birth control pills (14.4% vs. 7.7%, respectively) and condoms (18.8% vs. 9.0%, respectively).” But the authors did not test if these differences were statistically significant. Please rephrase or test each category.

**Discussion**

33. “Our study findings suggest that physical IPV during and around the time of pregnancy may impact postpartum contraceptive usage.” During and 12 months prior to pregnancy. The authors did not look at the postpartum period. Please clarify.
34. “Women who experienced physical IPV at the time of pregnancy” Please be consistent with terms.
35. “This is a significant issue, since IPV is associated with the risk of unintended pregnancy, sexually transmitted diseases, and other reproductive health concerns.” Yes, however, for these outcomes to occur you have to have exposure to sex. We see in Table 2 that ¼ of people who reported physical IPV are abstinent. Another 10% are sterilized/with a partner who is sterilized. It’s very difficult to know from this analysis if any of these people are actually at risk of unintended pregnancy and STDs.
36. “Comparable findings have been documented in earlier studies, such as in an analysis of PRAMS data from 2004 to 2008 found that women reporting IPV were significantly less likely to use postpartum contraception than women who did not (cOR, 0.66; 95% CI, 0.61-0.71) [31].”
    a. Please see: Stevenson AA, Bauman BL, Zapata LB, Ahluwalia IB, Tepper NK. Intimate Partner Violence around the Time of Pregnancy and Postpartum Contraceptive Use. Womens Health Issues. 2020 Mar-Apr;30(2):98-105. doi: 10.1016/j.whi.2019.11.006. Epub 2020 Jan 5. PMID: 31911042.
    b. This is the most up-to-date analysis with PRAMS data on this topic and should be incorporated.
37. “Our analyses indicated that women who experienced IPV had significantly lower usage rates for partner-dependent contraceptive methods such as condoms, natural family planning (including rhythm), 272 and vasectomy compared to their counterparts” . Where are these findings? The authors did not test if these different were statistically different from each other (Table 2).
38. “Our study found that women who have experienced physical IPV are more likely to rely on natural contraceptive methods-which is concerning as natural methods are less reliable and can be more difficult to use effectively, thus increasing exposure to unplanned pregnancy.” So in the previous sentence, the authors just said that there analysis indicated that IPV exposed women has less usage of partner dependent methods (rhythm, withdrawal, abstinence). Thus, this sentence contradicts that. This is also misleading because most of these women were using abstinence – meaning they do not have sex and thus cannot get pregnant. If you aren’t having sex, then you can’t be exposed to unplanned pregnancy.
    a. Also, withdrawal is 78% effective. Fertility awareness methods range in effectiveness anywhere from low 70% to high 90%. Abstinence is 100% effective. The pill/condom/ring/etc. can also be difficult to use correctly, which is why, for example, typical effectiveness of condoms is only 87%. This is much higher than withdrawal, but withdrawal is still better than “nothing” and it’s great if it fits someone’s values and preferences at that point in time.
39. “The fact that nonuse of postpartum contraception is higher, and possibly increasing, among women who have experienced physical IPV...” The Stevenson et al. (2020) paper found that 21% of women exposed to IPV were not using contraception. This study found that 9% of women exposed to IPV were not using contraception. In terms of trends, it looks like postpartum contraceptive use has improved among women exposed to physical IPV.
40. Even among women not exposed to IPV, 94% is high. Last year, an analysis of Phase 8 PRAMS data was published on postpartum contraceptive use. They found that about 15% of people did not use contraception. So this study found an even smaller percentage of nonuse.
    a. Bruce K, Stefanescu A, Romero L, Okoroh E, Cox S, Kieltyka L, Kroelinger C. Trends in Postpartum Contraceptive Use in 20 U.S. States and Jurisdictions: The Pregnancy Risk Assessment Monitoring System, 2015-2018. Womens Health Issues. 2023 Mar-Apr;33(2):133-141. doi: 10.1016/j.whi.2022.10.002. Epub 2022 Dec 1. PMID: 36464580.
41. “To address this issue, healthcare providers should adhere to recommended screening guidelines for IPV during pregnancy, and provide family planning, education, and resources to all patients, regardless of their disclosure.” Screening, alone, would likely not eliminate the effect of physical IPV on PPFP. However, preventing violence from occurring in the first place possibly could.
42. “In conclusion, our study underscores the importance of assessing IPV with the need of contraceptive methods as an important reproductive health outcome.” But this analysis didn’t consider whether women were actually screened and if that screening made a difference in contraceptive use. It doesn’t underscore the importance, it just shows that there is a relationship between IPV exposure and PPFP. There are many different interventions; screening could be one of them. Although, screening alone is not recommended by ACOG, USPSTF, or the CDC. These organizations recommend that IPV screening should always be coupled with referral and on-going support. It is likely that the referral and ongoing support make the difference, not screening alone.
43. “For example, those who participated in PRAMS were asked only a few general questions about IPV.” They asked exactly 2 questions.
44. “In addition, the term “abused” used in this survey, may be regarded as demeaning or judgmental”. The term “abused” is not used in the survey. The survey questions are:
    a. “In the 12 months before you got pregnant with your new baby, did any of the following people push, hit, slap, kick, choke, or physically hurt you in any other way?”
    b. “During your most recent pregnancy, did any of the following people push, hit, slap, kick, choke, or physically hurt you in any other way? “
45. Conclusion: again, screening alone will likely not impact reduced exposure to physical IPV or mediate this relationship with PPFP. There are a number of interventions that CDC, ACOG, and other organizations recommend to prevent violence before it occurs, especially for people who are pregnancy and postpartum.
    a. Suggest reading: https://www.cdc.gov/violenceprevention/pdf/ipv-technicalpackages.pdf
