# Supplementary material for: Association between physical intimate partner violence and postpartum contraceptive use in the United States–evidence from PRAMS 2016–2021
Source: PLoS One. 2024 Dec 11;19(12):e0314938. doi: 10.1371/journal.pone.0314938 (PMC11633987; doi:10.1371/journal.pone.0314938)
Supplement: Attachment — Submitted filename: Response to Reviewers_19April2024.docx [file pone.0314938.s003.docx]

April 19, 2024

**RE: PONE-23-33931**

Association between intimate partner violence and postpartum contraceptive use in the United States – Evidence from PRAMS 2016-2021

PLOS One

**Journal requirements:**

1. When submitting your revision, we need you to address these additional requirements.

Please ensure that your manuscript meets PLOS ONE's style requirements, including those for file naming. The PLOS ONE style templates can be found at 
https://journals.plos.org/plosone/s/file?id=wjVg/PLOSOne_formatting_sample_main_body.pdf and 
<https://journals.plos.org/plosone/s/file?id=ba62/PLOSOne_formatting_sample_title_authors_affiliations.pdf>.

This manuscript follows PLOS One’s style requirements, including those for file naming according to the shared templates.

2. Please provide additional details regarding participant consent. In the ethics statement in the Methods and online submission information, please ensure that you have specified what type you obtained (for instance, written or verbal, and if verbal, how it was documented and witnessed). If your study included minors, state whether you obtained consent from parents or guardians. If the need for consent was waived by the ethics committee, please include this information.

Once you have amended this/these statement(s) in the Methods section of the manuscript, please add the same text to the “Ethics Statement” field of the submission form (via “Edit Submission”).

For additional information about PLOS ONE ethical requirements for human subjects research, please refer to <http://journals.plos.org/plosone/s/submission-guidelines#loc-human-subjects-research>.

We have mentioned these issues in the ‘PRAMS Data’ and ‘Ethics approval’ section in this manuscript.

3. Note from Emily Chenette, Editor in Chief of PLOS ONE, and Iain Hrynaszkiewicz, Director of Open Research Solutions at PLOS: Did you know that depositing data in a repository is associated with up to a 25% citation advantage (https://doi.org/10.1371/journal.pone.0230416)? If you’ve not already done so, consider depositing your raw data in a repository to ensure your work is read, appreciated and cited by the largest possible audience. You’ll also earn an Accessible Data icon on your published paper if you deposit your data in any participating repository (<https://plos.org/open-science/open-data/#accessible-data>).

This study analyzed Pregnancy Risk Assessment and Monitoring Survey (PRAMS) data received from the U.S. Centers for Disease Control and Prevention (CDC). The data sources used in the analysis can be accessed at the weblink upon request: <https://www.cdc.gov/prams/prams-data/researchers.htm>.

4. Please include your full ethics statement in the ‘Methods’ section of your manuscript file. In your statement, please include the full name of the IRB or ethics committee who approved or waived your study, as well as whether or not you obtained informed written or verbal consent. If consent was waived for your study, please include this information in your statement as well.

Our ethic statement provided in the method section of this study, page 8, lines 102 - 111: The PRAMS Phase 8 (2016-2021) data is released to the public and is provided for use by the approval of PRAMS sites and CDC over request [21]. Researchers requested de-identified PRAMS data by submitting a proposal to CDC with a data sharing agreement, and a standard application form. The study protocol was approved by the CDC and PRAMS participating state health departments by informed written consent to data request email on December 1, 2022, and the data was shared within four weeks after this approval. Ethical approval of this study was waived by the UNC Chapel Hill Office of Human Research Ethics Institutional Review Board IRB (Study# 22-2814) in a written consent on January 23, 2023, because the study was carried out using publicly available data that was anonymized and free of personally identifiable information. Only members of the research team listed on the application had access to the data.

**Reviewers' comments:**
Review for PLOS One

**Reviewer #1:**

1. Suggest clarifying that this paper is about physical IPV in the title, as PRAMS also measures emotional and sexual IPV: “Association between intimate partner violence and postpartum contraceptive use in the United States– Evidence from PRAMS, 2016-2020”

We would like to thank the reviewer for this comment. In our study we focused on physical intimate partner violence (IPV) as this is covered by the core questionnaire. Therefor, in the revised manuscript we have updated the title as “Association between physical intimate partner violence and postpartum contraceptive use in the United States - Evidence from PRAMS 2016-2021”.

Abstract

2. Please be very clear on what type of IPV is the focus of this paper and please be consistent throughout with terminology. There is switching from IPV to physical IPV. I suggest also using the term physical IPV.

We would like to thank the reviewer for bringing out this point. We have corrected as required to be consistent with the terminology throughout the manuscript.

3. PRAMS uses the terminology phases, rather than rounds. It should be PRAMS Phase 8.

We would like to thank the reviewer for pointing out this. We have corrected the term as Phase 8 in this manuscript.

4. Why was 2021 data not included? That was released in April 2023.

We requested CDC PRAMS data from 2016 to 2020, but were in actuality provided data from 2016 to 2021.Thus, in our study all the calculations were done with PRAMS data 2016 to 2021. We have corrected the text and included a table on State-wise data collection for each year between 2016 to 2021 (Annex 1).

5. “Our sample included women who answered questions about the experience of physical IPV during and/or 12 months prior to their most recent pregnancy” – it’s not actually the most recent pregnancy. It’s the index birth from which the birth certificate was sampled. Someone could have had a pregnancy between the last birth and the timing of the survey.

We have updated this sentence to read, “Inclusion criteria ….. 165,204 women.” , page 5, lines 121-127.

6. “modern or natural method” how are you defining these categories? Why not just say use of any method?

We would like to thank the reviewer for your question. We have broadly categorized the methods of contraception as ‘modern method’ and ‘non-modern method’ (Hubacher & Trussel, 2015) to explain whether physical IPV has any impact in choosing postpartum contraceptive methods with the concept that women experiencing IPV may have different considerations and constraints when choosing between modern and non-modern methods. For instance, an abuser's control over a woman's autonomy may limit her access to healthcare services, thereby influencing her propensity to utilize natural methods over modern methods.

Hubacher D, Trussell J. A definition of modern contraceptive methods. *Contraception*. 2015;92(5):420-421. doi:10.1016/j.contraception.2015.08.008

7. This paper did not look at screening for physical IPV during pregnancy and/or its relationship to postpartum contraceptive use. The authors, however, suggest that “it is crucial to prioritize meeting unmet contraceptive needs as well as conducting universal screening for IPV among pregnant women.” Please align conclusions with the actual findings from this paper.

Thanks to the reviewer for pointing this out. We gave revised our suggesting as “It calls for the integration of IPV considerations into public health policies and clinical initiatives to improve maternal well-being.” In page 2, lines 33-34.

Introduction

8. “Research in the 1990s found that between 3% to 9% of pregnant women were estimated to have experienced IPV during pregnancy in the US, though the 63 real number could have been much higher than reported [13].” Why did the authors use this citation? These numbers were recently updated using PRAMS data…
   a. Please see: D'Angelo DV, Bombard JM, Lee RD, Kortsmit K, Kapaya M, Fasula A. Prevalence of Experiencing Physical, Emotional, and Sexual Violence by a Current Intimate Partner during Pregnancy: Population-based Estimates from the Pregnancy Risk Assessment Monitoring System. J Fam Violence. 2022 Jan 14;38(1):117-126. doi: 10.1007/s10896-022-00356-y. PMID: 37205924; PMCID: PMC10193455.

We would like to thank the reviewer for providing us with this reference. We have updated this sentence with references in page 3, lines 55-58.

9. “Mistimed or unwanted pregnancies can lead to induced abortion; an estimated 40% of unintended pregnancies end in abortion in the US [17]. Increases in correct and consistent contraceptive use, especially among populations at increased risk of having an unmet need for contraception, are therefore likely to lead to a decline in induced abortions.” Perhaps… but is this really a concern/goal? Induced abortion is not an adverse outcome and having an induced abortion could represent someone exerting their autonomy to do so and their reproductive preferences. Further, contraceptive use, alone, is not the solution to preventing unintended pregnancy. It is one tool that people can use to prevent pregnancy if that is their desire. I think this paper could use a good review of the framing to use more health equity/person centered language.

Thanks to the reviewer for bringing up this point. We have deleted these two sentences, and added the following sentence “It is anticipated that the incidence of unintended pregnancies could be reduced through the proper and consistent use of contraceptives, especially within populations with a higher risk of experiencing unmet needs for contraception.” in page 3, lines 65-67.

10. “However, to our knowledge, no recent studies have aimed at understanding the extent of the relationship of IPV on women’s contraceptive use in the US.” This is not true. The PRAMS team published an almost identical paper with data from Phase 7.
    a. Stevenson AA, Bauman BL, Zapata LB, Ahluwalia IB, Tepper NK. Intimate Partner Violence around the Time of Pregnancy and Postpartum Contraceptive Use. Womens Health Issues. 2020 Mar-Apr;30(2):98-105. doi: 10.1016/j.whi.2019.11.006. Epub 2020 Jan 5. PMID: 31911042.

Thank you for bringing this relevant publication to our attention. We appreciate your diligence in ensuring the accuracy of our statement regarding previous research on the relationship between intimate partner violence (IPV) and women’s contraceptive use in the United States.

We acknowledge the existence of the article by Stevenson et al., 2020. This study indeed explores a similar topic to ours, examining the impact of IPV on postpartum contraceptive use using data from Phase 7 of the Pregnancy Risk Assessment Monitoring System (PRAMS) data between 2012 to 2015 in 36 states and 1 city. Our data is more recent with the wider coverage of 46 states and 4 cites.

Methods

11. PRAMS is implemented in collaboration with state and local health departments

We would like to thank the reviewer for this comment. We have updated this information in page 4, line 84.

12. PRAMS isn’t always state level. NYC, DC and Puerto Rico are also included in Phase 8.

We have acknowledged this information in page 4, lines 86-87.

13. “These are the most recent available data containing information on IPV and contraceptive use behavior for women of reproductive age in the US” Why wasn’t 2021 data incorporated into this analysis? Then this sentence would be true.

With reference to our clarification made to the reviewer’s comment above (#4), we now state that the data included the period of 2015-2021 of Phase 8.

14. Again, please use the terminology that PRAMS prefer – Phase 8

Thanks to the reviewer. We have updated the manuscript with the terminology ‘Phase 8’ throughout.

15. To clarify, PRAMS does not collect data on gender identity. Many PRAMS publications use the term “women”, so I think it’s okay. However, a limitation is that we don’t actually know the gender identity of people included in this analysis, only that they were listed as the birthing parent on the birth certificate. So it is possible that people who do not identify as cisgender women were included and they are being misgendered by the terms used in this paper.

We appreciate the opportunity to address this important point. In response to this question, we included “Moreover, our analysis is based on individuals listed as the birthing parent on the birth certificates and may not fully encompass all gender identities.” as a limitation in our manuscript in page 16, lines 349-351.

16. It’s actually 2 – 6 months after delivery that people were mailed a survey.

Many thanks to the reviewer for pointing this out. We have corrected.

17. The introduction and justification for the study focuses heavily on use of contraception as a means to avoid unintended pregnancy. However, the authors did not restrict the study sample to only people who wanted to avoid a next pregnancy. I suggest that the authors either do this or add additional justification on why we would want people who want to achieve pregnancy use contraception. I think the authors are assuming this is the case, as the sample is people who gave birth in the prior 2-6 months, but many people in the US (30%) have interpregnancy intervals shorter than the recommended 18 months (although, 6 months is really the most adverse). I would suggest reframing in terms of why it is important women avoid short interpregnancy intervals.

To address this comment from the reviewer we re-wrote as “Post-partum contraception plays ………prevalence of unintended pregnancies.” In pages 3-4, lines 65-72.

18. PRAMS requires a minimum overall response rate threshold for the release of data for each year. Did all sites have data for all years included in this study? This should be specified. Some states only have data released for specific years, or even no years. So not all sites are included for every year available.

Several PRAMS sites have data released for specific years or may have no data available for certain years within our study period. In our revised manuscript, we have included a section in the methodology detailing the availability of data from each PRAMS site for the years included in our study.

In the manuscript, we included “A comprehensive list of available datasets for Phase 8 (year 2016-2021), categorized by city and state, is provided as an annex alongside this manuscript. The threshold for the minimum overall response rate was set at 55% for 2016 -2017, and 50% for 2018-2021.” in lines 105-108, pages 4-5 according to the reference (<https://www.cdc.gov/prams/prams-data/researchers.htm>).

19. Why was “other” contraceptive methods recoded? There are responses that do actually fit within the other categories because of data collection errors. I suggest recoding as many as possible.

The "other" contraceptive methods were recoded to ensure clarity and accuracy in analyzing the data.. From the category ‘other’ we have recoded as many answers as possible to the other categories of contraception, which did not make any changes to estimates due to the low number.

20. A huge limitation of using the method question is that lactational amenorrhea method (LAM) is not included. LAM does afford a good amount of protection from pregnancy. You will find some responses like this in the “other” response category.

Thank you for your valuable feedback regarding the omission of the lactational amenorrhea method (LAM) from the listed contraceptive methods in our study. The decision to not explicitly categorize LAM as a separate contraceptive method in our analysis was based on the constraints of the PRAMS questionnaire design and the categorization of responses. As you correctly pointed out, responses that might pertain to LAM use were likely captured under the "other" response category, which, unfortunately, does not allow for a detailed analysis of LAM's specific prevalence and impact.

In light of your comment, we have updated our manuscript adding in the limitation “LAM was not included …………….accessibility” On page 16, lines 358 – 363.

21. “This study does not have the scope to measure IPV beyond physical IPV as this information was not collected by the PRAMS core questions.” This is a study limitation; please move to discussion. Data is included on sexual and emotional IPV before, during, and after pregnancy, as well as physical IPV after pregnancy, but these are in the standard question set. You could have included this if you wanted to look at these associations.

This sentence was removed from the methods section.

In the limitation section of our manuscript, we explicitly discuss the limitation of not including these dimensions of IPV in our analysis, stating “We decided to focus on physical IPV in this study, because data on psychological and emotional abuse were not included for every state or city in the survey. “ in page 16, lines 351-352.

22. What was the justification for grouping together AI/AN, Asian, etc in “other”? From Table 1, 25,242 people were included in this group. A best practice is to maintain a minimum set of categories (American Indian or Alaska Native, Asian, Black or African American, Native Hawaiian or Other Pacific Islander, and White), except when the sample size is so small that estimates would be unreliable.

Thank you for your question regarding the grouping of American Indian/Alaska Native (AI/AN), Asian, and other racial/ethnic categories under the "other" designation in our manuscript. We appreciate your attention to detail and the opportunity to clarify our rationale for this categorization.

Given the relatively small sample sizes of AI/AN, Asian, and other racial/ethnic groups within the PRAMS dataset, combining these categories into a single group allowed us to maintain statistical power and precision in our analysis. This approach ensures that estimates for these smaller racial/ethnic groups remain reliable and robust, thus minimizing the risk of spurious findings due to small sample sizes.

While we acknowledge that a best practice is to maintain a minimum set of categories for reporting racial/ethnic data, we believe that our decision to combine AI/AN, Asian, and other racial/ethnic groups into the "other" category was justified within the context of our study objectives and the available sample size considerations, aligning with previous studies (Cha et al., 2015; Testa et al., 2023)

Cha S, Chapman DA, Wan W, Burton CW, Masho SW. Intimate partner violence and postpartum contraceptive use: the role of race/ethnicity and prenatal birth control counseling. *Contraception*. 2015;92(3):268-275. doi:10.1016/j.contraception.2015.04.009.

Testa A, Lee JG, Jackson DB, Mungia R, Ganson KT, Nagata JM. Physical intimate partner violence and prenatal oral health experiences in the United States. *BMC Oral Health*. 2023;23(1):749. Published 2023 Oct 12. doi:10.1186/s12903-023-03491-0

23. How were people classified if they selected more than one race?

People who selected more than one race were classified as ‘Other’ for analysis.

24. “The variable ‘state of residence’ was aggregated into four geographic 148 regions according to US Census divisions: Northeast, Midwest, South, and West [25].” Why? This likely absolves any association we would see between state of residence and access to contraception in the postpartum period.

We would like to thank the reviewer for this insightful question. We appreciate the opportunity to clarify our methodology behind the decision to aggregate states into four larger geographic regions was made for several reasons: firstly, this geographic representation allows for easier interpretation and comparison of our findings with existing literature and public health data (Snyder et al., 2020). Secondly, we aimed to reduce the potential confounding effects of individual state-level differences that may not be pertinent to the scope of this study.

Snyder JE, Stahl AL, Streeter RA, Washko MM. Regional Variations in Maternal Mortality and Health Workforce Availability in the United States. *Ann Intern Med*. 2020;173(11 Suppl):S45-S54. doi:10.7326/M19-3254

25. Why wasn’t insurance status included as a covariate?

While insurance status may be associated with both intimate partner violence and contraceptive use, the inclusion of this covariate could potentially introduce multicollinearity issues with other variables included in our analysis, such as socioeconomic status or access to healthcare services. In order to maintain the parsimony and interpretability of our model, we opted to focus on covariates that were most directly related to our research question and had the greatest potential impact on the outcomes of interest. Moreover, the requirements for Insurance coverage for using contraceptive methods varies largely from state to state (Ref: *https://www.guttmacher.org/state-policy/explore/insurance-coverage-contraceptives)*, which is beyond the scope of this study.

**Results**

26. “This contraceptive method-mix was calculated according to the efficacy of the last used method reported during the survey as described in the methods section.” This doesn’t need to be repeated.

This sentence is deleted.

27. Typo in this sentence: For the long-acting-reversible and permanent methods (LARC-PM) IUD and vasectomy use were hinger among IVP non-exposed group,”

Thanks to the reviewer for pointing this out. We have corrected the type ‘hinger’ to ‘higher’ in this sentence in line 237, page 9.

28. “we found that women who had exposure to physical IPV in the last twelve months had 42% lower odds” This phrasing is incorrect. It’s people who experienced IPV in the 12 months prior to pregnancy and during pregnancy. It’s not the last 12 months.

We thank the reviewer for suggesting this correction. We have updated our manuscript with this correction.

29. “The association between IPV and contraceptive use remained the same for modern contraceptive use only which was tested separately (results not shown).” I think a better comparison would be methods that require prescription/interaction with a facility/provider/etc and those that don’t. A likely explanation is that people who experience violence may not seek healthcare from a provider (either due to fears of confidentiality, lack of trauma informed/high quality care, barriers to getting care, etc), and so they might use methods that don’t require a prescription or interaction with the healthcare system. HOWEVER, some of the methods that the authors classified as “natural” require partner support… periodic abstinence, withdrawal, etc. But people using these methods might not use consistently or might not be as exposed to sex as people not experiencing violence. I assume that breastfeeding and sexual frequency might also differed between the groups, and these are factors for pregnancy… and these were not controlled for.

Your point about comparing contraceptive methods that require a prescription or interaction with healthcare providers versus those that do not is well taken. This perspective could indeed shed light on potential barriers to accessing certain types of contraception for individuals experiencing IPV, particularly in contexts where fear, confidentiality concerns, or lack of access to trauma-informed care may limit interactions with the healthcare system. Additionally, your observation regarding "natural" methods necessitating partner support, such as periodic abstinence and withdrawal, is also crucial, and is included in the discussion. These methods, while not requiring a healthcare provider's prescription, indeed depend on the cooperation and consent of both partners, which may not be reliably present in relationships characterized by IPV. This inconsistency can impact the effectiveness of such methods and the autonomy of the IPV survivor in reproductive decision-making.

Given these considerations, future research could incorporate a comparison of contraceptive methods based on the requirement for healthcare interaction and explore the hypothesis that IPV survivors may prefer methods with lower barriers to access. However, these issues are beyond the scope of the current study.

30. Table 2: “Natural contraceptive methods are rhythm, withdrawal, abstinence, other etc.” Please clarify what is “etc”.

We have categorized methods of contraception as modern and non-modern contraceptive methods (Hubacher & Trussell, 2015). Where, non-modern methods include rhythm, withdrawal, abstinence, and other. We have updated the footnote of defining non-modern methods in Table 2 by deleting “etc.”, in line 282, page 12.

31. Authors should clarify all abbreviations in tables with a note (e.g., IUD)

Thanks to the reviewers for this comment. We have updated the footnote of Table 2 with the abbreviation of IUD, in line 282, page 12.

32. “A main difference between women exposed to IPV and women who weren’t was in the use of birth control pills (14.4% vs. 7.7%, respectively) and condoms (18.8% vs. 9.0%, respectively).” But the authors did not test if these differences were statistically significant. Please rephrase or test each category.

In Table 2 we utilized Rao-Scott chi-square test to get results.

**Discussion**

33. “Our study findings suggest that physical IPV during and around the time of pregnancy may impact postpartum contraceptive usage.” During and 12 months prior to pregnancy. The authors did not look at the postpartum period. Please clarify.

We would like to thank the reviewer for pointing out this statement. We have corrected this statement as “Our study findings suggests that physical IPV during or within 12 months before pregnancy can have an impact on the use of postpartum contraception.” In page 14, lines 288-289. In this study, our outcome variable is the postpartum contraception use, and we are interested to investigate whether there is any association between postpartum contraception use and IPV.

34. “Women who experienced physical IPV at the time of pregnancy” Please be consistent with terms.

Thanks to the reviewer for pointing this out. In the revised manuscript we have changed it to “Women who experienced physical IPV with 12 months prior or during pregnancy ….” (page 14, lines 289-292), and we also have focused on maintaining consistency with these terms used in this study.

35. “This is a significant issue, since IPV is associated with the risk of unintended pregnancy, sexually transmitted diseases, and other reproductive health concerns.” Yes, however, for these outcomes to occur you have to have exposure to sex. We see in Table 2 that ¼ of people who reported physical IPV are abstinent. Another 10% are sterilized/with a partner who is sterilized. It’s very difficult to know from this analysis if any of these people are actually at risk of unintended pregnancy and STDs.

To address the reviewer’s concern regarding the actual risk of unintended pregnancy and STDs among the study population, given the reported rates of abstinence and sterilization among those who experienced IPV, we would like to do future research to further explore the nuances of reproductive health risks among IPV survivors, including the impact of abstinence and sterilization. This further research in future will help in understanding the diverse experiences and needs of this population can help tailor interventions and support services more effectively.

36. “Comparable findings have been documented in earlier studies, such as in an analysis of PRAMS data from 2004 to 2008 found that women reporting IPV were significantly less likely to use postpartum contraception than women who did not (cOR, 0.66; 95% CI, 0.61-0.71) [31].”
    a. Please see: Stevenson AA, Bauman BL, Zapata LB, Ahluwalia IB, Tepper NK. Intimate Partner Violence around the Time of Pregnancy and Postpartum Contraceptive Use. Womens Health Issues. 2020 Mar-Apr;30(2):98-105. doi: 10.1016/j.whi.2019.11.006. Epub 2020 Jan 5. PMID: 31911042.
    b. This is the most up-to-date analysis with PRAMS data on this topic and should be incorporated.

We would like to thank the reviewer for this comment. We have incorporated this updated citation in our manuscript.

37. “Our analyses indicated that women who experienced IPV had significantly lower usage rates for partner-dependent contraceptive methods such as condoms, natural family planning (including rhythm), 272 and vasectomy compared to their counterparts” . Where are these findings? The authors did not test if these different were statistically different from each other (Table 2).

In table 2, we have conducted bivariate analysis with IPV and method-mix using Rao-Scott χ^2^ tests (mentioned in the method section, page 7, line 181).

38. “Our study found that women who have experienced physical IPV are more likely to rely on natural contraceptive methods-which is concerning as natural methods are less reliable and can be more difficult to use effectively, thus increasing exposure to unplanned pregnancy.” So in the previous sentence, the authors just said that there analysis indicated that IPV exposed women has less usage of partner dependent methods (rhythm, withdrawal, abstinence). Thus, this sentence contradicts that. This is also misleading because most of these women were using abstinence – meaning they do not have sex and thus cannot get pregnant. If you aren’t having sex, then you can’t be exposed to unplanned pregnancy.
    a. Also, withdrawal is 78% effective. Fertility awareness methods range in effectiveness anywhere from low 70% to high 90%. Abstinence is 100% effective. The pill/condom/ring/etc. can also be difficult to use correctly, which is why, for example, typical effectiveness of condoms is only 87%. This is much higher than withdrawal, but withdrawal is still better than “nothing” and it’s great if it fits someone’s values and preferences at that point in time.

We deleted this sentence to avoid any confusion.

39. “The fact that nonuse of postpartum contraception is higher, and possibly increasing, among women who have experienced physical IPV...” The Stevenson et al. (2020) paper found that 21% of women exposed to IPV were not using contraception. This study found that 9% of women exposed to IPV were not using contraception. In terms of trends, it looks like postpartum contraceptive use has improved among women exposed to physical IPV.

The statement has been updated with “The fact that nonuse of postpartum contraception is higher, and possibly increasing, among women who have experienced physical IPV compared to their counterpart,……” in page 14, lines 298.

40. Even among women not exposed to IPV, 94% is high. Last year, an analysis of Phase 8 PRAMS data was published on postpartum contraceptive use. They found that about 15% of people did not use contraception. So this study found an even smaller percentage of nonuse.
    a. Bruce K, Stefanescu A, Romero L, Okoroh E, Cox S, Kieltyka L, Kroelinger C. Trends in Postpartum Contraceptive Use in 20 U.S. States and Jurisdictions: The Pregnancy Risk Assessment Monitoring System, 2015-2018. Womens Health Issues. 2023 Mar-Apr;33(2):133-141. doi: 10.1016/j.whi.2022.10.002. Epub 2022 Dec 1. PMID: 36464580.

We acknowledge the difference in findings across studies. Thanks to the reviewer for pointing out this issue. The methodological difference of our study with Bruce et al., (2023) was in the exclusion criteria for selecting data. They took only 20 sites from PRAMS for selecting their sample size for their study which they compared for four consecutive years to measure trend. Their trend analysis also shows non-use declining from 15.8 to 13.7 between 2018 to 2015, respectively. Since our data covers samples from 50 sites (including 46 states) it consists of greater power to accuracy. There could also be differences in definitions. We are not sure from their study, where they put ‘abstinence’ in defining types of contraceptive methods.

There could be contextual changes or interventions that might have occurred between the periods analyzed that could influence postpartum contraceptive use rates. We would propose further research to explore the discrepancies in findings and better understand trends in postpartum contraceptive use.

41. “To address this issue, healthcare providers should adhere to recommended screening guidelines for IPV during pregnancy, and provide family planning, education, and resources to all patients, regardless of their disclosure.” Screening, alone, would likely not eliminate the effect of physical IPV on PPFP. However, preventing violence from occurring in the first place possibly could.

Thanks to the reviewer for bringing this point. We have added in the revised manuscript “Screening by itself might not fully address the impact of physical IPV on postpartum contraceptive use. However, proactive violence prevention could potentially make a significant difference on this and other reproductive health outcomes.” In page 15, lines 319 – 320.

42. “In conclusion, our study underscores the importance of assessing IPV with the need of contraceptive methods as an important reproductive health outcome.” But this analysis didn’t consider whether women were actually screened and if that screening made a difference in contraceptive use. It doesn’t underscore the importance, it just shows that there is a relationship between IPV exposure and PPFP. There are many different interventions; screening could be one of them. Although, screening alone is not recommended by ACOG, USPSTF, or the CDC. These organizations recommend that IPV screening should always be coupled with referral and on-going support. It is likely that the referral and ongoing support make the difference, not screening alone.

We would like to thank the reviewers for their valuable comments. We have revised this statement as “Our research highlights ……………. health and wellbeing.” on page 15, lines 326 – 333.

43. “For example, those who participated in PRAMS were asked only a few general questions about IPV.” They asked exactly 2 questions.

We have updated this sentence with “For example, those who participated in PRAMS were asked only two questions about IPV.” on Page 15, line 322-323.

44. “In addition, the term “abused” used in this survey, may be regarded as demeaning or judgmental”. The term “abused” is not used in the survey. The survey questions are:
    a. “In the 12 months before you got pregnant with your new baby, did any of the following people push, hit, slap, kick, choke, or physically hurt you in any other way?”
    b. “During your most recent pregnancy, did any of the following people push, hit, slap, kick, choke, or physically hurt you in any other way? “

Thanks to the reviewer for this comment. We agree that the two questions to determine IPV do not contain the word ‘abuse’, but this section in the PRAMS questionnaire is named as “Abuse”; the these two questions are asked on page 5, line 1 (Reference: <https://www.cdc.gov/prams/pdf/questionnaire/Phase-8-Topics-Reference_508tagged.pdf> ). This is the reason we included this as a limitation of using PRAMS IPV questions.

45. Conclusion: again, screening alone will likely not impact reduced exposure to physical IPV or mediate this relationship with PPFP. There are a number of interventions that CDC, ACOG, and other organizations recommend to prevent violence before it occurs, especially for people who are pregnancy and postpartum.
    a. Suggest reading: <https://www.cdc.gov/violenceprevention/pdf/ipv-technicalpackages.pdf>

Thanks to the reviewer for pointing, it out and sharing the suggested reading. With reference to the suggested reading, we have updated the conclusion in the manuscript as ‘Postpartum women ……………….. health in the US.’ In page 16, lines 365 – 373.

**Reviewer #2:** The manuscript entitled, "Association between intimate partner violence and postpartum contraceptive use in the United States– Evidence from PRAMS, 2016-2020” is an important update on a topic that remains relevant. There are several aspects that commend this manuscript, including a generally clear writing style, and importance of topic. There are few minor typographical errors and some less than accurate phrasing. There are other considerations that should be examined prior to acceptance for publication. These are summarized below.

Lines 16-17 & 91: The PRAMS surveillance system is comprised of different “Phases” of the survey – not “rounds” as the authors state. As such, they utilized data from Phase 8 of the survey, not “round 8”.

We would like to thank the reviewer for pointing it out. We corrected “round” as “phase” in this study.

Line 59: In the phrase “In the US 1 in 5… it should be a numeral 1 and not the letter I.

Many thanks to the reviewer for this comment. We have corrected.

Lines 61-63: The authors cite research from the 90s. This discussion could have benefited from an examination of and comparison with more recent studies of a similar vein including the following.

1. Prevalence of Experiencing Physical, Emotional, and Sexual Violence by a Current Intimate Partner during Pregnancy: Population-based Estimates from the Pregnancy Risk Assessment Monitoring System.
D'Angelo DV, Bombard JM, Lee RD, Kortsmit K, Kapaya M, Fasula A.
J Fam Violence. 2022 Jan 14;38(1):117-126.

2. Intimate Partner Violence Before and During Pregnancy, and Prenatal Counseling Among Women with a Recent Live Birth, United States, 2009-2015.
Kapaya M, Boulet SL, Warner L, Harrison L, Fowler D.
J Womens Health (Larchmt). 2019 Nov;28(11):1476-1486.

Thanks to the reviewer for your comment and sharing references. We have updated with recent references as “A recent …. Reported.”, page 3, lines 55-58.

Lines 76-77: The authors state that to their “knowledge, no recent studies have aimed at understanding the extent of the relationship of IPV on women’s contraceptive use in the US.” This is incorrect. There are a few fairly recent studies that have done just that including the reference below which is only cited in the discussion portion of this paper. The authors could have introduced Stevenson et al, 2020 here and used it to describe if or how their work differs and how it adds to prevailing literature.

"Intimate Partner Violence around the Time of Pregnancy and Postpartum Contraceptive Use.
Stevenson AA, Bauman BL, Zapata LB, Ahluwalia IB, Tepper NK.
Womens Health Issues. 2020 Mar-Apr;30(2):98-105.

We upated this sentence as “There is no ….. in the U.S..” in page 4, lines 78-80.

Line 84: PRAMS is not an observational study. Please correct this.

We made necessary correction.

Line 96: The word “survey” is missing after PRAMS.

We included “survey” after PRAMS.

Lines 96--97: This sentence is inaccurately stated. PRAMS was not “designed to collect a stratified sample of mothers who recently gave birth at participating sites using data from birth certificates” rather, PRAMS utilizes a stratified sample pulled from birth certificate records of women with a recent live birth in participating sites, to collect data on their experiences and behaviors before, during, and shortly after pregnancy.

Thanks to the reviewer for this comment. We corrected this sentence as “The PRAMS survey employes a stratified sample extracted from birth certificate records of women who have recently given birth in participating sites.” On page 5, lines 101-102.

Lines 124-125: The wording used here does not accurately reflect the PRAMS question cited which in fact reads "Are you or your husband or partner doing anything now to keep from getting pregnant? " If the authors choose to paraphrase the question, quotation marks should not be used as though citing directly.

In the PRAMS core questionnaire two questions are asked on contraception. These two questions are “Are you or your husband or partner doing anything now to keep from getting pregnant?”, and if so, asked the following question “What kind of birth control are you or your husband or partner using now to keep from getting pregnant? “in page 6, lines 138-141.

Line 129: Please correct the typo in spelling of Mirena which is spelled “Minera”

Thanks to the reviewer. We made this correction.

Lines 134-135: The authors refence birth control methods here not previously listed in the methods they include in their outcome variable (lines 127-134), namely, tubectomy, vasectomy and abstinence.

Thanks to the reviewer for pointing it out. We have updated the outcome variable including all methods.

Lines 138-140: The PRAMS survey has emotional and sexual abuse questions on the standard questions list. Why did the authors not request these standard questions? As reported in D'Angelo et al 2022, emotional IPV was the most frequently reported form of IPV, more prevalent than physical violence. It would have been beneficial to see if the patterns of contraceptive use differed by type of IPV.

We have not considered to include emotional IPV, covered in the standard questionnaire, because this questionnaire does not cover by all participated States, thus we’ll lose information.

Results

Line 178: Add the word ‘live’ before birth here (and anywhere else in the manuscript when describing the sample used.

Added accordingly.

Table 2, Lines 201-210: Again, the authors have included on the table and in the description of results, methods not referenced in the initial write up of the BC methods to be analyzed. They are reporting on tubectomy, vasectomy and abstinence which were never referenced in lines 127-134. Further, the authors introduce new terms in Table 2 “coil” and “loop” (types of IUD) not previously mentioned elsewhere. If these refer to the trade names of BC methods cited previously, they should either use coil and loop in parentheses against each respective trade name or remove the terms from the table and retain only the collective term “IUD” as used in their methods section.

We made the group of contraceptive methods consistent throughout the manuscript.

Discussion

Line 264: Authors cite a “worsening trend” in view of results from data from 2004-2008. How do their results compare against more recently published similar studies such as Stevenson AA et al 2020?
Also, it would be interesting for the authors to give their thoughts around the higher prevalence of abstinence reported among those with IPV exposure compared to those without which is driving the higher prevalence of natural contraceptive methods in this group compared to modern methods. Particularly given it is a partner-dependent method.

Thanks to the reviewer for this comment. We updated as “However, almost one-quarter ……. Noticeable contraceptive option.” In page 14, lines 309-313.

General comment: Finally, I would suggest clarifying throughout the paper that the variable under question is PHYSICAL IPV. This is explained in the methods quite nicely, but the results, discussion and tables should clearly indicate that this study applies to physical IPV only.

Thanks to the reviewer for pointing this out. Our paper in focused on ‘physical IPV’ which we clarified throughout the paper.
