# Supplementary material for: Association between physical intimate partner violence and postpartum contraceptive use in the United States–evidence from PRAMS 2016–2021
Source: PLoS One. 2024 Dec 11;19(12):e0314938. doi: 10.1371/journal.pone.0314938 (PMC11633987; doi:10.1371/journal.pone.0314938)
Supplement: Attachment — Submitted filename: Responses to Reviewers_13Sept2024.docx [file pone.0314938.s005.docx]

August 31, 2024

**RE: PONE-23-33931**

Association between intimate partner violence and postpartum contraceptive use in the United States – Evidence from PRAMS 2016-2021

PLOS One

Minor Revisions Required

Review comments

General comment: Thank you for responding to my previous comments. I still have concerns after reading the revision. I am flagging for the editor that some revisions were not made in the manuscript text. I am also noting some issues with the framing of this paper. Please see my notes below. I believe this paper could benefit from a thorough review of the US literature on the relationship between IPV and contraceptive use (with a focus in the postpartum period). I also believe this paper could be reframed using a reproductive justice lens, rather than some of the instrumental arguments it seems to be using as justification.

This paper is actually opportune to call attention to the ways in which violence, specifically physical IPV, plays a role in people’s ability to have children, not have children, and raise children in safe and sustainable environments. It draws on almost no perspectives from the violence prevention literature as well, which I see as a huge flaw. If IPV is associated with PPFP, then the conclusions should reflect that and call to invest in programming to reduce and prevent IPV before it occurs, and perhaps offer some evidence-based interventions or way to intervene and prevent IPV (so that postpartum people can make decisions with full autonomy).

Specific comments:

1. We have broadly categorized the methods of contraception as ‘modern method’ and ‘non-modern method’ (Hubacher & Trussel, 2015) to explain whether physical IPV has any impact in choosing postpartum contraceptive methods with the concept that women experiencing IPV may have different considerations and constraints when choosing between modern and non-modern methods. For instance, an abuser's control over a woman's autonomy may limit her access to healthcare services, thereby influencing her propensity to utilize natural methods over modern methods. Hubacher D, Trussell J. A definition of modern contraceptive methods. Contraception. 2015;92(5):420-421. doi:10.1016/j.contraception.2015.08.008
   a. Hubacher & Trussell (2015) is more applicable to the international context. Additionally, their classification of lactational amenorrhea method (LAM) as “non modern” is outdated and conflicts with newer classification systems that recognize LAM as a highly effective method for postpartum people. The same is true for standard days method (SDM) which is also considered a modern method in the context of LMICs (and I believe in the US). I suggest the authors draw on US literature, considering the sample is US postpartum people.
   b. The above rational that the authors provide does not consider that “nonmodern” methods like withdrawal require a tremendous amount of support from male partners. Likewise, past research suggests that fertility awareness-based methods (FABs) also require partner support, because often they require the partner to agree to periods of monthly abstinence. Some modern methods can also be used covertly. There is an entire body of literature on covert use by women experiencing IPV. I suggest the authors reframe to the US context, and again suggest the authors consider any method of family planning considering that the goal of FP programs and services is to support people in using whatever method matches their values and preferences, not just modern methods.
   c. I am also noting that the authors seemed to upload a version of their response to the reviews with comments. I see in the comments the authors noting that: “Modern” family planning (FP) methods refer to the following: pill, intrauterine device, implant, injectable, condom (male and female), spermicide, diaphragm, patch, vaginal ring, sponge, cervical cap, lactational amenorrhea method, emergency contraception, standard days method, basal body temperature method, TwoDay method, sympto-thermal method, and sterilization (tubal ligation and vasectomy).” Why wasn’t this definition of modern methods used? I believe it is much more consistent with the US literature and classification systems.
   d. I am also flagging that there is a focus on use on modern contraception… but then the results seem to be about any method of contraception? This is very confusing for the reader. I would suggest being clear about what is the focus of this paper.

We would like to thank the reviewer for the detailed discussions on the categorization of contraceptive methods. After reviewing the results, we have decided to drop the analysis comparing use of modern with non-modern, and keep only ‘any method’ to continue with the paper.

Therefore, we have deleted “However, women exposed to physical………. Opposite trend (56% and 69%, respectively).” From abstract from page 1, lines 24-26.

Deleted “These contraceptive methods ……… for the analysis.” In page 6, lines 149 to 156 form the method section.

Additionally deleted “The use of modern ….. level (*p*<0.001).” in page 10, lines 250 to 255, and “The association …… tested separately.” In page 11, lines 288 to 289 from the result section.

We also have deleted ‘categories of contraceptive methods’ from Table 2.

2. “Thanks to the reviewer for bringing up this point. We have deleted these two sentences, and added the following sentence “It is anticipated that the incidence of unintended pregnancies could be reduced through the proper and consistent use of contraceptives, especially within populations with a higher risk of experiencing unmet needs for contraception.” in page 3, lines 65-67”
   a. I am flagging that I did not find this sentence in the manuscript. I am flagging for the editor to make sure that all changes that the authors reported were in fact reflected in the paper.

We would like to thank the reviewer for flagging the absence of this sentence. We apologize that this sentence was revised in the manuscript, but not updated in the rebuttal. The updated sentence was “It is anticipated that the effective and consistent use of contraceptives has the potential to decrease the unmet need for postpartum contraception, particularly among populations with higher prevalence of unintended pregnancies [18].” In page 4, lines 69-72.

b. This new sentence needs a citation. Please also define what populations are at higher risk of experiencing unmet need for contraception in the US context.

We have included reference for this revised statement with the following citation,:

Schummers L, Hutcheon JA, Hernandez-Diaz S, et al. Association of Short Interpregnancy Interval With Pregnancy Outcomes According to Maternal Age. *JAMA Intern Med.* 2018;178(12):1661–1670.

Here, we have defined the population as ‘who had higher prevalence of unintended pregnancies’.

3. “The rationale is to provide information for policy makers and stakeholders in planning and implementing policies to boost utilization of postpartum contraceptives to improve maternal health with specific focus on the physical IPV exposed women.”
   a. How does use of contraception improve maternal health in the US context? This is not clear from this sentence of the preceding text about unintended pregnancy and nonuse of contraception. In the US, recent studies have shown that the evidence is even mixed on the casual effect of short interpregnancy intervals on some maternal health outcomes. ACOG provides a good overview here: <https://www.acog.org/clinical/clinical-guidance/obstetric-care-consensus/articles/2019/01/interpregnancy-care#:~:text=Because%20the%20interpregnancy%20period%20is,a%20continuum%20from%20postpartum%20care>.

We would like to thank to the reviewer for this comment. We have revised the statement as “The rationale of this study is to inform policymakers and stakeholders in planning and implementing policies aimed at improving reproductive and maternal health, with a specific focus on enhancing postpartum contraceptive use among women exposed to physical IPV in the United States.” in page 4, lines 80 to 83.

b. Why would or should the goal of PPFP programs and services be to increase uptake of postpartum contraception? If the goal is to improve maternal health and/or reduce violence (as the authors state), there are numerous, effective interventions that can improve the availability and quality of maternal and infant health services and a number of strategies that can be implemented proven to prevent IPV in the US.

As noted above, the text referring to increasing uptake of PPFP was removed.

4. “Post-partum contraception plays a crucial role in safeguarding women’s health by effectively preventing short interpregnancy intervals… minimizing associated health complications [9,15].”
   a. This sentence seems to have been added in the update. References 9 and 15 are about the relationship between violence and reproductive health broadly. If the authors want this sentence included, they should provide a citation to support claims that postpartum contraception prevents short interpregnancy intervals in the US context.

We would like to thank the reviewer for this comment. We included this sentence in the previous revised version. Here we have updated the citation.

5. “Moreover, our analysis is based on individuals listed as the birthing parent on the birth certificates and may not fully encompass all gender identities.””
   a. I appreciate that the authors added this as a limitation, but I think it’s missing the issue. The analysis cannot establish gender identity because the survey does not ask about gender identity. Thus, it cannot be assumed that it is only cis-gender women in this analysis. It could be that there are some transmen or other individuals of diverse genders included in this study, and so use of the term “woman/women/mothers” may not accurately represent the study population. In addition, findings and conclusions cannot be generalized to LGBTQ+ populations. It is also difficult to disentangle and account for infertility in this population. Some respondents state that they used ART to become pregnant (either because of infertility or because of same sex relationships) in open-ended questions. However, the analysis doesn’t account for that and so there may be some people who are not using family planning because they are not at risk of pregnancy.

We would like to thank the reviewer for this detailed comment. We used the term ‘woman/women/mother’ in our manuscript to represent the sample population of this survey who had given birth between 2 and 6 months before the survey was conducted. The PRAMS questionnaire also uses term ‘women’ in their questionnaire/topic reference document (reference is given below).

PRAMS Phase 8 Topic Reference Document. Weblink: <https://www.cdc.gov/prams/pdf/questionnaire/Phase-8-Topics-Reference_508tagged.pdf>

Moreover, previous articles using PRAMS data have also used the term ‘women’ explaining for their sample population in their manuscripts (Kapaya et al., (2019); Alhusen et al., (2015)).

Alhusen, J. L., Ray, E., Sharps, P., & Bullock, L. (2015). Intimate partner violence during pregnancy: maternal and neonatal outcomes. *Journal of women's health (2002)*, *24*(1), 100–106. <https://doi.org/10.1089/jwh.2014.4872>

Kapaya, M., Boulet, S. L., Warner, L., Harrison, L., & Fowler, D. (2019). Intimate Partner Violence Before and During Pregnancy, and Prenatal Counseling Among Women with a Recent Live Birth, United States, 2009-2015. *Journal of women's health (2002)*, *28*(11), 1476–1486. <https://doi.org/10.1089/jwh.2018.7545>

b. “The PRAMS survey employes a stratified sample extracted from birth certificate records of women who have recently given birth in participating sites. This sample is used to collect data on their experiences and behaviors before, during, and shortly after pregnancy. Women were mailed a questionnaire within 2–6 months following delivery, and those who did not respond to repeated mailings were contacted by 105 telephone. Participants were consented prior to completing the PRAMS survey per CDC protocols [21].”
   i. Here is an example pulled from the methods. The authors state that the sample is drawn from birth certificate records of women who recently have a given birth. Gender identity is not asked in birth certificate records. It is the person who was recorded as the infant’s mother that was sampled. We actually don’t know how this person identifies, so by the authors calling them “women” it could be misgendering people. Similarly, it is people who gave birth, not necessarily “women”, who were mailed the questionnaire.

To address this comment, we have included “Moreover, our analysis is based on individuals listed as the birthing parent on the birth certificates and may not fully encompass all gender identities.” as a limitation in our manuscript in page 16, lines 349-351 as a limitation in our manuscript.

ii. Also, it’s a sample of live births.

Thanks to the author for pointing this out. We have corrected it.

6. I appreciate the rationale for race/ethnicity groups that was provided in the response. However, it is best practice to provide the sample size for each group. Please provide rationale for the race/ethnicity categories (Black non-Hispanic, White non-Hispanic, all other races non-Hispanic, and Hispanic) in the manuscript text with sample sizes for all the race categories that were collapsed into other.

We would like to thank the reviewer for this comment. We have updated race/ethnicity as “race/ethnicity (categorized as ‘Black non-Hispanic’, ‘White non-Hispanic’, all other races (e.g., Other Asian (4.3%), American Indian (3.6%), Chinese (1.2%), Japanese (0.2%), Philipino (0.8%), Hawaiian (0.1%), Ak Native (0.8%), mixed race (5.7%), and other non-White (5.5%))” in page 7, lines 175-176. In the result section we have added the all the race categories that were collapsed into ‘other’ in page 9, lines 229 to 231.

7. I appreciate the rationale for the groups of states. I advise the authors to review the PRAMS methodology, as well as PRAMS publications. It is common for PRAMS analysis to include each individual state. Also, it did not come through in reading the paper that the authors wanted to make claims about geographic differences. “this geographic representation allows for easier interpretation and comparison of our findings with existing literature and public health data”. Please add these interpretations to the results section. This would greatly benefit the paper if that was an objective of the analysis. Please also add this justification to the methods section.

We would like to thank the review for these comments. In the methodology section we also included “with the aim to reduce the potential confounding effects of individual state-level differences that many not be pertinent to the scope of this study.” After categorizing state of residences in page 7, lines 174-175. Findings on regional variation are presented for Figure 2.

We have added “Moreover, the prevalence of physical IPV varies significantly across different states in the USA. Some states have more robust support systems and legal protections for IPV survivors, which can influence reporting rates and overall prevalence of IPV [41].” In the discussion section, in page 16, lines 351 to 353.

8. The rationale for exclusion of insurance status is not clear. Did the authors test insurance status for multicollinearity with other covariates? I don’t see this in the methods or in the response to reviewers.

The decision not to include insurance status as a covariate was based on the direct acyclic graph (DAG) used in the study design. The DAG identified a minimally sufficient adjustment set of covariates that were considered essential for controlling confounding in the relationship between intimate partner violence (IPV) and postpartum contraceptive use. The focus of this study is on demographic, socioeconomic, and reproductive health variables that were theoretically and empirically supported as significant confounders based on prior literature. Insurance status was not identified as a necessary covariate within this context and was therefore not included in the final model. This decision also aligns with the aim of creating a simplified model to avoid excessive adjustments, which could undermine statistical power and lead to biased estimates.

9. “Household income in the 12 months before last live childbirth was categorized as ‘less than or equal to median household income’ or ‘above median household income’ compared to the 2021 US Census median income level of $71,186”
   a. I am now noticing that income was created compared to the 2021 US census income level. However, data is from 2016-2021. Other PRAMS analysis have created a poverty scale based on the HHS Poverty Guidelines for that year. I would suggest a similar methodology, especially if household income is the proxy for health insurance, or add justification for only basing income off 2021.

Thanks to the reviewer for this comment. We have revised the household income level from the HHS Poverty Guidelines considering the average median household income for year 2016-2022. In the revised manuscript it has been updated as “Household income in the 12 months before last live childbirth was categorized as ‘less than or equal to the average median household income’ or ‘above average median household income’ created from the poverty scale based on the HHS Poverty Guidelines for years 2016-2021 [28]. On average, the median household income over 2016-2021 was approximately $69,878 [28].” In page 7, lines 183-186.

10. “Thanks to the reviewer for this comment. We agree that the two questions to determine IPV do not contain the word ‘abuse’, but this section in the PRAMS questionnaire is named as “Abuse”; the these two questions are asked on page 5, line 1 (Reference: https://www.cdc.gov/prams/pdf/questionnaire/Phase-8-Topics-Reference_508tagged.pdf ). This is the reason we included this as a limitation of using PRAMS IPV questions.”
    a. The topical reference guide is not the survey. This is a guide for researchers who are using the survey, and so the questions are categorized by topic. Here is the survey: [Pregnancy Risk Assessment Monitoring System (PRAMS) Phase 8 Topic Reference Document (cdc.gov)](https://www.cdc.gov/prams/pdf/questionnaire/Phase-8-Topics-Reference_508tagged.pdf)
    b. Just in case, here are the questions in the survey and text:

“Pregnancy can be a difficult time.. The next questions are about things that may have happened before and during your most recent pregnancy.

In the 12 months before you got pregnant with your new baby, did any of the following people push, hit, slap, kick, choke, or physically hurt you in any other way? For each person, check No if they did not hurt you during this time or Yes if they did. No Yes a. My husband or partner * * b. My ex-husband or ex-partner * * c. State option (Another family member) * * d. State option (Someone else) * * Insertion point for Standard question Z14 29. During your most recent pregnancy, did any of the following people push, hit, slap, kick, choke, or physically hurt you in any other way? For each person, check No if they did not hurt you during this time or Yes if they did. No Yes a. My husband or partner * * b. My ex-husband or ex-partner * * c. State option (Another family member) * * d. State option (Someone else) * * Insertion point for Standard question Z1 9 Phase 8 Core Mail Questionnaire June 2016 AFTER PREGNANCY The next questions are about the time since your new baby was born.”

We would like to thank the reviewer for this comment. We dropped the following text about limitations, “In addition, the term “abused” used in this survey, may have negative connotations, impacting participants responses [41]. Several projects have subsequently demonstrated that many women who initially denied being ‘abused’ but later admitted to experience physical violence [42].” from our revised manuscript. In pages 16-17, lines349-351.

11. “These data represent the most recent available information on physical IPV and contraceptive use behavior among women of reproductive age in the United States.”
    a. This is not true. Phase 9 is out which is now the most recent. Please exclude or explain that Phase 9 is the most recent data.

We agree that Year 2022 (Phase 8) PRAMS data is released already, therefore we have updated this sentence as “This is the largest data representing the information on physical IPV and postpartum contraceptive use behavior among women of reproductive age in the United States.”

12. “From the unadjusted models, we found that exposure to physical IPV in the last 12 months, mother’s age, race-ethnicity, region, urban/rural residence, marital status, employment, and intention of last pregnancy were statistically significant predictors of postpartum contraceptive use without adjusting for other covariates”
    a. The variable was not “physical IPV in the last 12 months”. It was physical IPV during pregnancy or 12 months before pregnancy. Please address this throughout the paper and please be consistent with terms. For example, in other parts of the paper, it is called “physical IPV before or during their last pregnancy”.
    b. In table 3, “IPV in the past 12 months”

We would like to thank the reviewers for this comment. We addressed this term as “physical IPV during pregnancy or 12 months before pregnancy” throughout the paper.

13. “Women who experienced physical IPV within 12 months prior or during pregnancy were significantly less likely to use postpartum contraception compared to those who did not report physical IPV, even after controlling for factors associated to contraceptive use. This is an important issue, as physical IPV is associated with the risk of unintended pregnancy, sexually transmitted diseases, and other reproductive health concerns among sexually active post-partum women [33].”
    a. I am not sure how these sentences are connected. The second sentence seems to come from nowhere. The focus of this paper was the relationship between physical IPV and contraceptive use. It would be good to relate back to the topic of interest. The authors could emphasize that we know that IPV is related to a number of adverse sexual and reproductive health outcomes, and this research builds on this evidence based and continues to demonstrate that physical IPV experienced 12 months before or during pregnancy contributes to nonuse of contraception in the postpartum period.

We would like to thank the reviewer for the comment. We agree that the connection between the two sentences could be clearer. We have revised the text as follows: “Women who experienced physical IPV during that period were significantly less likely to use postpartum contraception compared to those who did not report physical IPV during the same period; even after controlling for factors associated with post-partum contraceptive use. This finding aligns with existing evidence that physical IPV is associated with adverse sexual and reproductive health outcomes, such as unintended pregnancy, sexually transmitted diseases, and other reproductive health concerns among sexually active post-partum women in the United States [33]. Our research builds on this evidence by demonstrating that physical IPV is associated with nonuse of contraception in the postpartum period, further highlighting the public health importance of addressing IPV in maternal health interventions.” In page 15, lines 296-304.

This revision emphasizes how our findings fit within the broader context of IPV’s impact on reproductive health while maintaining a clear focus on the study’s key outcome.

14. “However, our findings suggest a worsening trend.”
    a. A worsening trend in what? Physical IPV? This paper didn’t analyze trends in physical IPV or postpartum contraceptive use.

We would like to thank the reviewer for pointing this out. We made correction as “Our findings suggest that the likelihood of postpartum contraceptive use among women who have experienced physical IPV has since decreased.” in page 15, lines 321-322.

15. “The reduced usage of these methods could be attributed to partner negotiation, potentially resulting in higher rates of nonuse, as suggested elsewhere [34].”
    a. Where is elsewhere? In the United States?

Yes, we mentioned the name of report, in page 15, line 308.

16. “The increased prevalence of abstinence and non-modern contraceptive methods among individuals who have experienced IPV may indicate a lack of reproductive autonomy and limited healthcare access, leading to a dependence on partner-controlled and less noticeable contraceptive option.”
    a. Please provide citations for this sentence. It could also be a sign that people are asserting their autonomy by deciding not to have sex or using traditional methods that match their values and preferences.

We would like to thank the reviewer for this comment. We have decided to delete this statement from our manuscript. In page 15 – 16, lines 334 – 337.

b. Also, increased prevalence as compared to what? Previous time periods?

We would like to thank the reviewer for this comment. We rewrote the sentence with citation as “This disparity in circumstance may underscore the complex interplay between intimate partner violence (IPV), the dynamics of power within relationships, and the decision-making processes regarding reproduction, thereby highlighting the necessary for interventions that consider these broader contexts [38].” In page 15, lines 322-325.

17. “Though our analysis did not evaluate the effectiveness of screening practices directly, our findings align with recommendations from ACOG 331 [9,38], USPSTF [39], and CDC [3], which emphasize the importance of integrating screening with referral and support services to address the impact of physical IPV on reproductive health outcomes”
    a. The analysis doesn’t support recommendations for screening. It supports the assumption that physical IPV may impact SRH decisions, specially contraceptive use. And so, it may be beneficial to prevent IPV and reduce exposure to IPV around the time of pregnancy, so that people can use contraception if that is what they want.

According to your suggestion we rephrased the statement as, “Our research highlights the association between exposure to physical IPV during pregnancy or 12 months before pregnancy, and its influence on postpartum contraceptive use. This underscores the importance of preventing IPV to support women’s reproductive autonomy. Our findings also suggest that reducing IPV exposure around the time of pregnancy could empower women to make autonomous contraceptive decisions. Comprehensive IPV interventions, aligned with recommendations from ACOG [9,39], USPSTF [40], and CDC [3], may play a crucial role in promoting women’s overall health and well-being.” In page 16, lines 339-345.

18. “On a broader scale, clinical and policy initiatives aimed at enhancing maternal health in the US should incorporate IPV as a public health policy concern.”
    a. There are several policy and clinical recommendations that do address and recognize IPV as a significant public health problem for SRH and maternal and infant health in the US – some were mentioned in the discussion. I think I’m struggling with the surface level nature of the introduction and discussion. It would be good to reflect and think deeply about what this analysis does to improve our understanding of IPV as a SRH issue in the US. What does it tell us that we previously did not know? How can it meaningfully inform practice and policy right now, building on the policies that we do already have and the practices that are already in place? Maybe shifting the emphasis from “women should use contraception” to “we need to prevent IPV because it can have adverse effects for people and their health and wellbeing” might help.

We would like to thank the reviewer for this detailed review comments. According to your suggestions, we revised our conclusion as “The study findings reveal the impact of physical IPV on postpartum contraceptive use, highlighting the need to shift focus from encouraging contraception to preventing IPV, which is vital for ensuring women’s reproductive autonomy and well-being. These findings call for integrating IPV prevention into maternal health strategies and enhancing support for affected women, building on existing policies. Further research should continue to explore the long-term effects of IPV on maternal health to further inform clinical and policy initiatives.” in page 17, lines 376-381.
