# Supplementary material for: Association between physical intimate partner violence and postpartum contraceptive use in the United States–evidence from PRAMS 2016–2021
Source: PLoS One. 2024 Dec 11;19(12):e0314938. doi: 10.1371/journal.pone.0314938 (PMC11633987; doi:10.1371/journal.pone.0314938)
Supplement: Attachment — Submitted filename: Responses to Reviewers_14 Nov 2024.docx [file pone.0314938.s006.docx]

November 14, 2024

**RE: PONE-23-33931**

Association between intimate partner violence and postpartum contraceptive use in the United States – Evidence from PRAMS 2016-2021

PLOS One

Minor amendments Required
Thanks for conducting such a good research. I am concerned that under the conclusions more detailed recommendations are needed with greater specificity. Since IPV is the current burning question and the study affects reproductive rights of women, it requires more recommendations to solve the problem. I would be interested to know whether you are linked to the international human right to reproductive rights. Otherwise, this manuscript written in good English and adds value to the existing science.

Thank you for your thoughtful feedback and for recognizing the value of our research. We truly appreciate your suggestions and agree that providing more detailed and specific recommendations in the conclusions section is crucial, especially given the importance of addressing IPV and its impact on reproductive rights.

The updated conclusion of the paper is written as “The study findings reveal the impact of physical IPV on postpartum contraceptive use, highlighting the need for IPV screening during pregnancy including the provision of person-centered post-partum family planning education and counseling, which is vital for ensuring women’s reproductive autonomy and well-being. These findings also call for integrating IPV prevention into maternal health strategies and enhancing support for affected women, building on existing policies. Further research should continue to explore the long-term effects of IPV on maternal health to inform clinical and policy initiatives further.” As echoing the detailed recommendation we mentioned in the discussion section of this manuscript.
